# Hypoxia induces polycystin-1 expression in the renal epithelium

**DOI:** 10.1098/rsos.220992

**Published:** 2023-05-17

**Authors:** Steffen Grampp, Andre Kraus, Kathrin Skoczynski, Mario Schiffer, René Krüger, Stephanie Naas, Johannes Schödel, Bjoern Buchholz

**Affiliations:** Department of Nephrology and Hypertension, Uniklinikum Erlangen and Friedrich-Alexander-Universität Erlangen-Nürnberg, Erlangen, Germany

**Keywords:** polycystic kidney disease, PKD1, transcription regulation, hypoxia, hypoxia-inducible factor, kidney development

## Abstract

Mutations in polycystin-1 which is encoded by the *PKD1* gene are the main causes for the development of autosomal dominant polycystic kidney disease. However, only little is known about the physiological function of polycystin-1 and even less about the regulation of its expression. Here, we show that expression of *PKD1* is induced by hypoxia and compounds that stabilize the hypoxia-inducible transcription factor (HIF) 1*α* in primary human tubular epithelial cells. Knockdown of HIF subunits confirms HIF-1α-dependent regulation of polycystin-1 expression. Furthermore, HIF ChIP-seq reveals that HIF interacts with a regulatory DNA element within the *PKD1* gene in renal tubule-derived cells. HIF-dependent expression of polycystin-1 can also be demonstrated *in vivo* in kidneys of mice treated with substances that stabilize HIF. Polycystin-1 and HIF-1*α* have been shown to promote epithelial branching during kidney development. In line with these findings, we show that expression of polycystin-1 within mouse embryonic ureteric bud branches is regulated by HIF. Our finding links expression of one of the main regulators of accurate renal development with the hypoxia signalling pathway and provides additional insight into the pathophysiology of polycystic kidney disease.

## 1. Introduction

Autosomal dominant polycystic kidney disease (ADPKD) is the most common monogenic renal disease often leading to end-stage renal disease [1]. ADPKD is characterized by the development of multiple bilateral renal cysts which originate from the tubular system [2]. Continuous enlargement of the cysts leads to compression of adjacent intact nephrons, which then results in a decline of renal function [2]. Mutations in the gene *PKD1*, which encodes for polycystin-1 (PC1), are the main causes for the development of ADPKD [3]. The underlying mechanisms leading to the development of cysts owing to *PKD1* mutations are incompletely understood. This may primarily be referred to the fact that the physiological role of PC1 has remained elusive so far. PC1 is an 11 transmembrane domain-containing glycoprotein, which is widely expressed in epithelial cells, vascular smooth muscle, cardiac myocytes and many other cell types [4]. In addition, PC1 shows varying subcellular localization which underlies a complex regulation of trafficking and various physical and functional interactions [4]. PC1 has a large extracellular region which has homology to domains that are involved in protein–protein or protein–carbohydrate interactions [5]. The short intracellular C-terminal tail interacts with the corresponding region of polycystin-2/TRPP2 [6]. The complex of PC1-TRPP2 is localized in the membrane of the primary cilium where its function is still a matter of debate [7].

In ADPKD, continuous cyst growth is accompanied by regional tissue hypoxia in the kidneys, which leads to upregulation of hypoxia-inducible transcription factor (HIF-1*α*) in cyst-lining cells [8]. HIF-1*α* then promotes cyst enlargement by transcriptional activation of target genes which mediate calcium-activated chloride secretion like the purinergic receptor P2Y2R whose stimulation leads to activation of the chloride channel TMEM16A [9]. Under physiological conditions, oxygen sensing and consecutive adaptation of cellular functions to changes in oxygen concentration is a pivotal necessity for cell survival. In the presence of molecular oxygen, the α-subunit of HIF is hydroxylated at specific proline residues by prolyl-hydroxylases (PHD), which then enables binding to the von Hippel–Lindau (VHL) protein [10]. The VHL protein mediates recognition of HIF by a ubiquitin E3 ligase complex, which targets hydroxylated HIF to proteasomal degradation [10]. In the absence of molecular oxygen, HIF-α remains stable and binds to its constitutively expressed ß-subunit [11]. The heterodimer translocates into the nucleus, where it acts as a transcription factor by binding to specific hypoxia response elements (HRE), which leads to transcriptional activation of many hundreds of target genes [12,13]. These genes are involved in metabolic adaptation, angiogenesis, cell cycle regulation, apoptosis and epithelial transport [13]. A second layer of regulation is mediated by the asparaginyl hydroxylase factor inhibiting HIF (FIH), which hydroxylates an asparagine residue within the *α* subunits and reduces interaction with the transcriptional machinery, thereby reducing HIF activity [14].

There exist two main isoforms of HIF-α, HIF-1*α* and HIF-2*α*, which show partly overlapping but also distinct target gene regulation [15]. In the kidney, HIF-1*α* is expressed in tubular epithelial cells, and HIF-2*α* in glomerular and peritubular cells, as well as VHL-defective clear cell renal cancer cells, which derive from the proximal tubule [16]. The physiological oxygen gradient within the kidney results in activation of HIF-2*α* in peritubular fibroblasts, which mediates renal erythropoietin production [16]. In adult healthy kidneys, however, HIF-1*α* is hardly expressed in tubule epithelial cells [17]. By contrast, during kidney development which takes place under hypoxic conditions, HIF-1*α* is expressed in ureteric branching epithelial cells and promotes nephron endowment [18]. During the course of various renal diseases, HIF-1*α* may become upregulated leading to beneficial but also devastating effects [13].

Given the importance of oxygen gradients for the regulation of target genes in the kidney during development and its impact on the course of renal diseases, we wondered if *PKD1* expression may be influenced by hypoxia. Therefore, we used human primary tubular epithelial cells (hPTC), wild-type mice for *in vivo* approaches, and *ex vivo* cultured embryonic mouse kidneys to test for the impact of hypoxia and HIF-1*α* on PKD1 RNA and PC1 protein expression.

## 2. Results

### 2.1. PC1 expression is regulated by hypoxia and HIF-1*α* in human primary tubular epithelial cells

**Figure 1.**
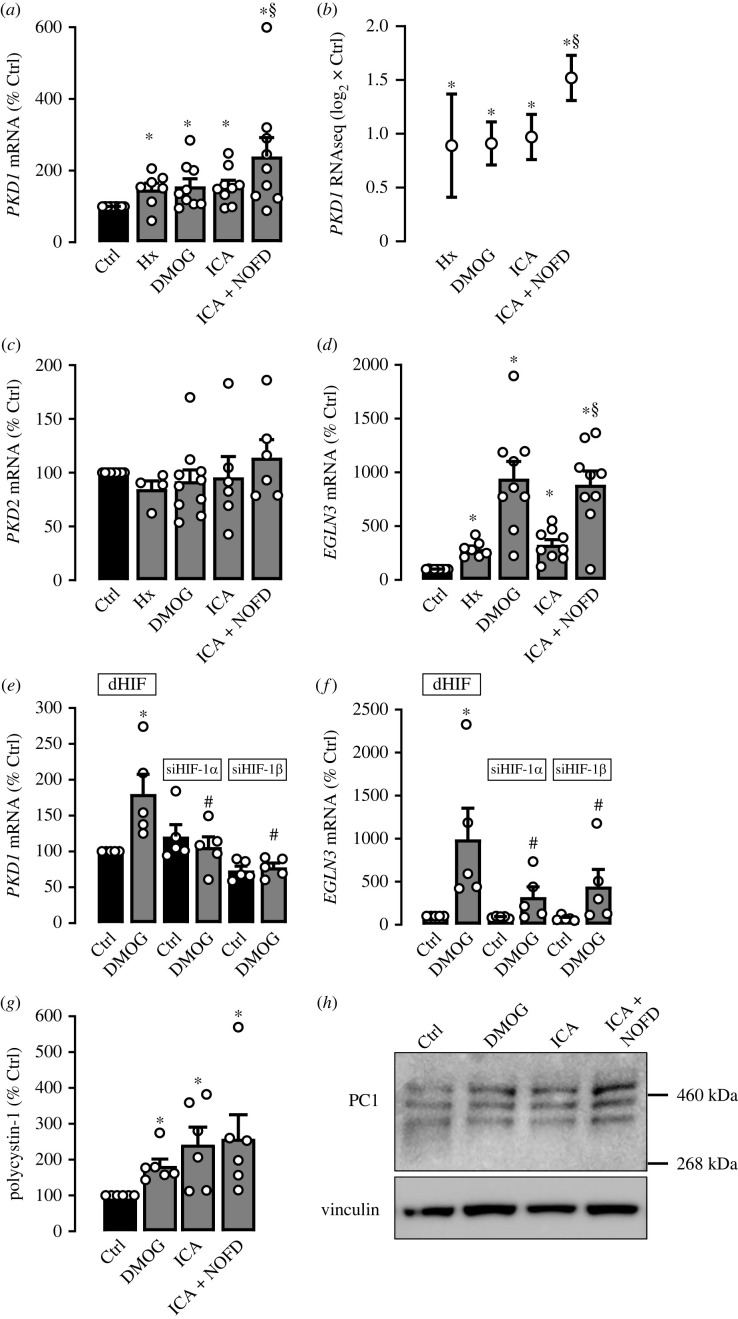
*PKD1* is a target gene of HIF-1*α* in human primary renal tubular cells. Human primary epithelial tubular cells (hPTC) from *n* = 9 individuals were cultured under control conditions (Ctrl) or exposed to hypoxia (Hx; 1% O_2_) or incubated with the inhibitors of HIF-α prolyl-4-hydroxylase domain (PHD) enzymes dimethyloxalylglycine (DMOG; 1mM) or 2-(1-chloro-4-hydroxyisoquinoline-3-carboxamido) acetate (ICA; 100 µM) for 16 h. In addition to ICA, Dimethyl N-oxalyl-D-phenylalanine (NOFD; 1mM), an inhibitor of the HIF-α asparagine hydroxylase factor inhibiting HIF (FIH) was applied. (*a*) *PKD1* mRNA expression in the presence of hypoxia, DMOG, ICA or ICA+NOFD in comparison with control (set = 100%). (*b*) *PKD1* RNAseq data from hPTC exposed to hypoxia (*n* = 3), or incubated with DMOG (*n* = 7), ICA (*n* = 3) or ICA in combination with NOFD (*n* = 3) expressed as log_2_-fold change ± the standard error of log_2_-fold change compared with control. (*c*) The same cells as described in (*a*) were tested for *PKD2* mRNA expression in the presence of hypoxia, DMOG, ICA or ICA+NOFD in comparison with control (set = 100%). (*d*) Cells described in (A and (*c*) were tested for HIF-1α-regulated *EGLN3* mRNA expression serving as positive control for HIF activity. (*e*) hPTC from *n* = 5 individuals were incubated with DMOG (1 mM) in the presence and absence of siRNA directed against HIF-1*α*, HIF-1*β* or *Drosophila* HIF (dHIF) serving as control condition. *PKD1* mRNA expression is depicted in comparison with control cells under untreated control condition (set = 100%). (*f*) EGLN3 mRNA expression of the cells described in (*e*) was analysed in comparison with control cells in the presence of siRNA directed against *Drosophila* HIF (set = 100%). (*g*) hPTC were supplemented with control medium (Ctrl), the PHD-inhibitors DMOG or ICA or ICA in addition to the FIH-inhibitor NOFD. Polycystin-1 protein expression from *n* = 6 individuals normalized for vinculin was analysed in comparison with control (set = 100%). (*h*) Representative Western blot from (*g*) with vinculin serving as loading control. * significant compared with control; # significant compared with DMOG; § significant compared with ICA. Wilcoxon signed-rank test (*a,c,d,g*), one-way ANOVA (*e*,*f*), Benjamini–Hochberg method within DESeq2 (*b*).

In order to explore regulation of PC1 by hypoxia we used freshly isolated hPTC and exposed them to 1% O_2_ for 16 h. We measured a moderate but significant increase of *PKD1* mRNA levels to 1.5 ± 0.18 fold by qPCR in cells exposed to hypoxia when compared with standard culture conditions (figure 1*a*). Similar results were obtained with RNA-seq (figure 1*b* and electronic supplementary material, file S1). Of note, chemical stabilization of HIF with dimethyloxalylglycine (DMOG) or 2-(1-chloro-4-hydroxyisoquinoline-3-carboxamido) acetate (ICA), both of which are known inhibitors of PHD enzymes, led to a robust induction of PKD1 mRNA in hPTC (figure 1*a*). Exposing cells to dimethyl N-oxalyl-D-phenylalanine (NOFD), an inhibitor of the HIF-α asparagine hydroxylase FIH and thus an activator of HIF transcriptional activity [19], in combination with ICA further increased PKD1 mRNA expression compared with cells treated with ICA alone (figure 1*a*). Again, RNA-seq data confirmed *PKD1* regulation by HIF-stabilization (figure 1*b* and electronic supplementary material, file S1). By contrast, mRNA expression of *PKD2* whose mutation also leads to ADPKD, was not affected by hypoxia or pharmacological HIF-stabilization (figure 1*c*). *EGLN3*, a well-characterized direct target gene of HIF served as positive control (figure 1*d*). We next used siRNA directed against human HIF-1*α*, the HIF-α isoform expressed in tubule cells, or its dimerization partner HIF-1*β* to compare the effects obtained by application of DMOG with control-treated hPTC (figure 1*e*). DMOG resulted in a significant increase of *PKD1* mRNA expression in control-treated cells, whereas *PKD1* mRNA expression was not induced in cells transfected with siRNA directed against HIF-1*α* or HIF-1*β* (figure 1*e*). Again, *EGLN3* served as positive control (figure 1*f*). This indicates HIF-1α-dependent regulation of *PKD1* on a transcriptional level in hPTC. In addition to mRNA regulation, we tested for effects on PC1 protein expression. Therefore, we treated hPTC with DMOG, ICA, or ICA and NOFD. Each treatment led to a significant elevation of PC1 protein level compared with control condition (figure 1*g,h*). We detected the three characteristic bands for PC1 in the Western blots, which have been described previously [20]. These represent (i) the mature full-length protein (PC1-FL), (ii) the EndoH-resistant N-terminal fragment (PC1-NTR), and (iii) the immature EndoH-responsive N-terminal PC1 fragment (PC1-NTS) which has not yet crossed the Golgi apparatus [20]. Similar results for protein quantification were obtained using Amido black instead of vinculin as loading control (electronic supplementary material, figure S1*a*,*b*). Stabilization of HIF-1*α* was confirmed by Western blot using both vinculin and Amido black as loading controls (electronic supplementary material, figure S1*c*–*e*).

These data suggest HIF-1α-dependent regulation of *PKD1* in human primary tubular epithelial cells.

### 2.2. HIF interacts with an intragenic regulatory DNA element of PKD1

**Figure 2.**
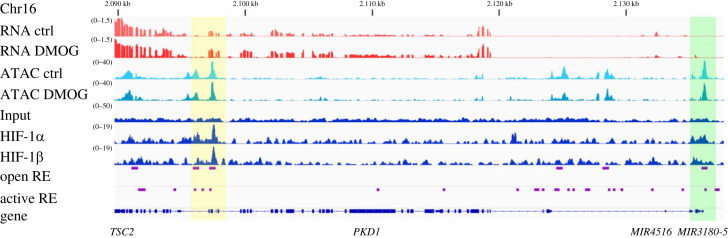
HIF-1 binds to an intragenic region of *PKD1*. HIF-1*α* and HIF-1*β* ChIP-seq tracks reveal HIF DNA interactions (yellow box) in an intragenic region approximately 40 kb upstream of the *PKD1* transcriptional start site (green box) in hPTC in which HIF was stabilized by 1 mM DMOG. Sequencing data for the input DNA is shown as a control. RNA-seq tracks indicate expression of PKD1 mRNA and regulation by DMOG. Data are representative and were generated from cells isolated from the kidney of one individual. Open chromatin at the *PKD1* locus is present at the promoter and the HIF-binding sites as determined by ATAC-seq in hPTC. Open and active regulatory (defined by chromHMM analysis) elements (RE) from human kidney samples overlap the HIF-binding site in hPTC.

In order to verify direct regulation of *PKD1* by HIF, we screened available HIF ChIP-seq data from hPTC. Inspecting the gene locus of *PKD1*, we identified HIF-1*α* and HIF-1*β* binding events in an intragenic region approximately 40 kb upstream of the *PKD1* transcriptional start site (figure 2). HIF-binding at this locus was associated with increased chromatin accessibility defined by ATAC-seq in the same cell type (figure 2). Furthermore, we defined accessible chromatin regions in the human kidney using published ATAC-seq data from kidneys of 15 individuals (provided by the Susztak laboratory and downloaded from https://cmdga.org/). This confirmed accessibility of the HIF-binding sites in kidneys from a greater cohort and supports the potential of this site to act as a regulatory element in kidneys *in vivo*. We also performed chromHMM analysis to define active chromatin regions in the kidney using published genome-wide screens of histone markers in kidney tissue [21,22]. This revealed features of an active regulatory element for the intragenic HIF-binding sites in whole kidney tissue (figure 2). To further validate our results, we confirmed HIF-1*α* and HIF-1*β* binding by ChIP-qPCR in samples from DMOG-treated hPTC isolated from three additional individuals (electronic supplementary material, figure S2). Furthermore, we resorted to publicly available HIF ChIP-seq data generated in a variety of renal tubule-derived cell lines including HKC-8, a proximal tubule-derived cell line, and RCC4 and 786-0 cells, which are VHL-defective clear cell renal cancer cell lines with proximal tubule origins [23,24]. In these cells, we detected conserved DNA interactions of HIF-subunits with the putative enhancer corroborating our findings from the hPTC ChIP experiments (electronic supplementary material, figure S3*a*). Confirming our data of hypoxic *PKD1* regulation in hPTC, we also detected upregulation of *PKD1* mRNA in hypoxic HKC-8 cells in a published RNA-seq dataset (electronic supplementary material, figure S4*a*). Interestingly, inspecting the *PKD1* enhancer in the index of human DNaseI hypersensitive chromatin sites defined by Meuleman *et al*. [25] from 733 biosamples revealed that this site coincides with accessible chromatin only in two components: (i) renal/cancer and (ii) organ development/renal (electronic supplementary material, figure S3*b*). By contrast, the promoter region of *PKD1* displayed comparable levels of accessibility across the different components (electronic supplementary material, figure S3*b*). This indicates the potential existence of a renal cell-specific enhancer at this position which mediates HIF-dependent *PKD1* regulation.

### 2.3. HIF-1 is the dominant HIF-isoform regulating PKD1 expression

We also observed binding of HIF-2*α* in HKC-8, RCC4 and 786-0 cells in the published ChIP-seq data and wondered whether this isoform could contribute to *PKD1* regulation under certain circumstances, e.g. in renal cancer, when HIF-2*α* is released by defective VHL protein (electronic supplementary material, figure S3*a*). 786-0 cells do not express functional HIF-1*α*, thus in these cells HIF-2 is the sole effector of the HIF transcriptional response. We identified published RNA-seq experiments from 786-0 cells, in which expression of HIF was manipulated in different ways [24,26]. First, in HIF-2*α* knockout 786-0 cells or 786-0 cells treated with the HIF-2 inhibitor PT2385, levels of *PKD1* mRNA did not change compared with untreated control cells, suggesting that HIF-2 does not regulate *PKD1* transcription (electronic supplementary material, figure S4*b*,*c*). Levels of *CCND1* and *EGLN3* mRNA, well-characterized HIF-2 targets in these cells, were significantly reduced by both measures (electronic supplementary material, figure S4*b*,*c*). The phenomenon that HIF-2*α* interacts with DNA, but does not necessarily trans-activate gene expression has been observed at a variety of loci [27,28]. Second, only overexpression of HIF-1*α*, but not HIF-2*α* led to a significant induction of *PKD1* mRNA in 786-0 cells (electronic supplementary material, figure S4*d*,*e*). These results indicate that in renal cancer cells, in which HIF-2*α* is present, it does not substantially contribute to *PKD1* expression.

Thus, expression of *PKD1* is mainly regulated by interactions of HIF-1 with an intragenic regulatory element in human renal epithelial-derived cells.

### 2.4. Pharmacological induction of HIF in mice results in increased expression of PKD1 mRNA and PC1 protein in the kidneys

**Figure 3.**
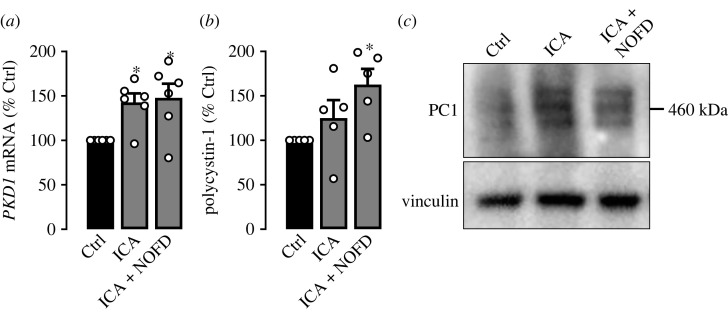
Pharmacological induction of HIF in mice results in increased expression of *PKD1* mRNA and PC1 protein in the kidneys. C57BL/6 mice were either intraperitoneally injected with the PHD-inhibitor ICA (*n* = 6; 40 mg kg^−1^), the combination of ICA and the FIH-inhibitor NOFD (*n* = 6; 80 mg kg^−1^) or vehicle (*n* = 6; Ctrl). (*a*) *PKD1* mRNA expression in comparison with control (set = 100%) from whole kidney lysates after 6 h exposure to the indicated treatment. (*b*) Polycystin-1 protein expression normalized for vinculin in comparison with control (set = 100%) from whole kidney lysates (*n* = 5 per condition). (*c*) Representative Western blot from (*b*) with vinculin serving as loading control. * significant compared with control; § significant compared with ICA. Wilcoxon signed-rank test.

We proceeded to analyse HIF-dependent regulation of *PKD1* in the kidney *in vivo*. Consequently, we treated C57BL/6 mice with ICA or ICA in combination with NOFD by intraperitoneal injection. After 6 h whole kidneys were harvested and tested for *PKD1* mRNA expression (figure 3*a*). In line with our previous findings, ICA treatment and the combination of ICA and NOFD resulted in a significant increase of PKD1 mRNA expression (figure 3*a*). In concordance with the mRNA data, HIF stabilization by ICA alone or in combination with FIH-inhibition, resulted in an increase of PC1 protein expression in whole kidney lysates reaching statistical significance in the combination of ICA and NOFD (figure 3*b*,*c*). Again, similar results for protein quantification were obtained using Amido black instead of vinculin as loading control (electronic supplementary material, figure S5*a*,*b*). Stabilization of HIF-1*α* in mouse kidneys was confirmed by Western blot and the use of vinculin and Amido black as loading controls (electronic supplementary material, figure S5*c–e*).

These data complement the *in vitro* data and confirm HIF-dependent regulation of *PKD1* also *in vivo* in mouse kidneys.

### 2.5. PC1 is expressed in ureteric bud branching epithelial cells during kidney development, and expression is increased under HIF-stabilizing conditions

**Figure 4.**
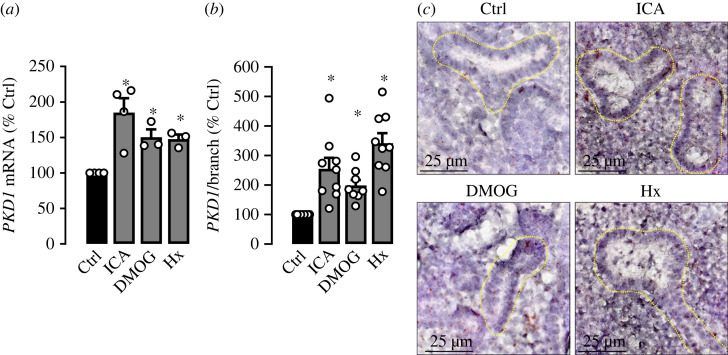
*PKD1* mRNA is expressed in ureteric bud branching epithelial cells during kidney development and is regulated in a HIF-dependent manner. C57BL/6 metanephric kidney pairs from embryonic day 13.0 were cultured *ex vivo*. One kidney was kept under control condition whereas the contralateral kidney was either incubated with the PHD-inhibitor ICA (100 µM; *n* = 4) or DMOG (1 mM; *n* = 3) or kept under hypoxic conditions (Hx; 1% O_2_; *n* = 3) for 24 h. (*a*) *PKD1* mRNA expression in comparison with control (set = 100%) from whole kidney lysates. (*b*) Wild-type mouse metanephric kidney pairs from embryonic day 13.0 were cultured *ex vivo* for 24 h. One kidney was kept under control condition and the contralateral kidney was either exposed to ICA (100 µM) or DMOG (1 mM) or 1% O_2_ (Hx). Thereafter, *in situ* hybridization for *PKD1* mRNA was performed, and expression was quantified within ureteric bud branches defined as the ratio of *PKD1* positive area to total branch area (*n* = 8–10 branches per kidney and condition) and analysed in comparison with control (set = 100%). (*c*) Representative *in situ* hybridization stainings of *PKD1* mRNA within ureteric bud branches (highlighted by green dotted lines) under control condition and in the presence of ICA, DMOG or hypoxia (Hx). * significant compared with control. Wilcoxon signed-rank test.

Recently, we have shown that HIF-1*α* promotes ureteric bud branching during mouse kidney development [18]. PC1 has also been shown to be involved in branching morphogenesis of embryonic kidneys [29]. Therefore, we wanted to analyse the effect of HIF on PC1 expression in metanephric kidneys. Metanephric kidneys from C57BL/6 mice were harvested at embryonic day (E) 13.0 and cultured *ex vivo*. One kidney was kept under control condition whereas the contralateral kidney was stimulated with the HIF-stabilizer ICA or DMOG or cultured under hypoxic conditions (1% O_2_). ICA, DMOG and hypoxia resulted in a significant increase of PKD1 mRNA in metanephric kidney lysates (figure 4*a*). HIF-1*α* induction was confirmed by a significant increase of mRNA of the HIF-1*α* target gene glucose transporter 1 (*GLUT1*) under HIF-stabilized conditions (electronic supplementary material, figure S6*a*). Again, as shown in human primary cells, *PKD2* mRNA expression was unaffected by HIF-stabilization (electronic supplementary material, figure S6*b*). We also applied *in situ* hybridization using RNAscope technique to define the sites of PKD1 mRNA expression. This revealed subtle expression of *PKD1* mRNA within ureteric bud branches under control conditions (figure 4*b*,*c*). However, exposure of the metanephric kidneys to ICA, DMOG or hypoxia resulted in a significant increase of PKD1 mRNA expression within but not restricted to the ureteric bud branches (figure 4*b*,*c* and electronic supplementary material, figure S6*c*).

These data confirm that PC1 is expressed in ureteric bud branching epithelial cells during mouse kidney development and that its expression depends on HIF-stabilizing conditions.

## 3. Discussion

PC1 is a large 11 transmembrane domain-containing protein with various sequences potentially serving as extracellular receptor, co-acting as a complex with TRPP2 to mediate ion conductance, and regulating cell–cell contacts and proper function of the primary cilium [4,30]. Loss of PC1 function results in ADPKD, which, due to the localization of PC1 (and TRPP2) in the primary cilium, is classified as a ciliopathy [31]. However, given the broad range of potential involvements in cellular functions and the severe consequences caused by PC1 dysfunction, only little is known about its precise physiological role(s). Considering the significant impact of PC1 on cell function, it seems likely that PC1 expression needs a precise regulation allowing the cell to adapt to physiological or pathophysiological changes. In addition, PC1 expression has been shown to significantly vary during development, further suggesting complex transcriptional regulation [32]. However, up to now, only very little is known about transcriptional regulators of *PKD1*. Potential regulatory elements have been identified within the promoter of *PKD1*, and regulatory potential has been supposed by cell-based reporter assays for the transcription factor E2F, as well as myeloid zinc finger (MZF), zinc finger protein (ZBP), and protein C-ets-1 (Ets1), and for one of three E-box containing elements [33]. Mutation of the E2F3 site confirmed transcriptional regulation of *PKD1 in vitro* indicating calcium-dependent effects on *PKD1* transcription [33]. However, there has been no insight into the transcriptional regulation of *PKD1 in vivo*.

Here, we show that *PKD1* is transcriptionally regulated by HIF-1*α* in epithelial cells *in vitro* and by HIF-stabilization *in vivo*. HIF-1*α* is the master regulator of cellular adaptation to changes in oxygen supply [34]. It affects a plethora of signalling pathways comprising cell–cell contact, cell proliferation and apoptosis, and is also linked to ciliary signalling pathways [35]. Although this does not prove functional involvement of PC1 in any of these HIF-mediated pathways, it is not surprising that expression of PC1 with its potentially multi-functional properties on different cellular behaviours is affected by HIF-1*α*. Of note, we focused on the expression of *PKD1* in renal epithelial cells and therefore mainly discuss hypoxia- and HIF-stabilizer-dependent regulation of *PKD1* as being mediated by HIF-1*α*, which is the prominent HIF-α isoform in these cells. However, when using whole kidney lysates, it seems possible that non-tubular cells via HIF-2*α* also contributed to increased *PKD1* levels.

HIF-1*α* is significantly involved in epithelial branching events during embryonic development which is a prerequisite for the proper development of organs like the kidneys, lungs and salivary glands [36]. In line with our data, PC1 has been shown to be significantly expressed in these organs, especially at those times when branching takes place. PC1 is particularly found in and around those cells that undergo branching such as in ureteric bud cells in the kidney and the surrounding condensing mesenchyme [29]. In the adult kidney, PC1 is mainly expressed in the collecting duct, which is physiologically exposed to low oxygen levels [29]. Therefore, it is intriguing to speculate that at least parts of the HIF-mediated effects in these organs during development could be caused by PC1. This idea is further strengthened by our finding that PC1 expression is increased in metanephric kidneys by pharmacological induction of HIF. PC1 has been shown to act in concert with TRPP2 [37]. This is mostly supported by the fact that mutations of both PC1 and TRPP2 lead to ADPKD [30]. However, during development, PC1 and TRPP2 show distinct expression patterns [38]. In the kidney, in contrast to PC1, TRPP2 is not expressed in the condensing mesenchyme and mainly localized to the tubular epithelium of the cortical region from murine development to adulthood [38]. Again, speculative, but the differences in expression could be referred to the hypoxia-responsiveness of *PKD1*, whereas expression of TRPP2 is not affected by hypoxia in hPTC (figure 1*c*).

ADPKD kidneys, at least at later stages of the disease, are characterized by regional hypoxia leading to upregulation of HIF-1*α* in the cyst epithelium [8]. At these stages, we have shown that HIF-1*α* is detrimental by further promoting cyst growth, particularly by activation of calcium-activated chloride secretion [9]. By contrast, at much earlier stages, activation of HIF-1*α* might be beneficial if it had the potential to increase at least partially functional PC1 expression, which would depend on the underlying mutations. This is of significant interest, since PHD inhibitors, which lead to activation of HIF, have been approved as erythropoiesis stimulating agents (ESA), which we fear may be detrimental in later stages of the disease. By contrast, PHD inhibitors could be beneficial at earlier stages (which usually would not be the typical time point to initiate ESA therapy) [13].

In conclusion, our findings provide insight into the transcriptional regulation of *PKD1*, which depends on HIF-1*α* and therefore on cellular oxygen supply and may be prone to unintended regulation by PHD inhibitors, which represent a new class of ESAs. Being aware of the HIF-dependent regulation may help to unravel the function of PC1 under physiological and pathophysiological conditions.

## 4. Material and methods

### 4.1. Cell culture

Human primary tubular epithelial cells (hPTC) from male and female patients were isolated from renal cortical tissues collected from healthy parts of tumour nephrectomies. Isolated cells represent a mixture of proximal and distal tubular cells with a ratio of approximately 50 : 50%, as described previously [39]. Isolation of human cells was approved by the local ethics committee (Reference number 542_20 Bc, Ethik-Kommission der Medizinischen Fakultät der Friedrich-Alexander Universität Erlangen-Nürnberg). Cortex tissue was cut into 1 mm^3^ pieces and digested with collagenase type II (Gibco, Karlsruhe, Germany) and DNase I grade II (Roche Diagnostics, Mannheim, Germany) for 60 min. Then, cell suspension was sieved through 100 and 70 µm meshes, and cells were seeded in epithelial cell selective medium (DMEM/Ham's F12 medium containing 2 mM L-glutamine, 100 U ml^−1^ penicillin, 100 mg ml^−1^ streptomycin, insulin-transferrin-selenium supplement, 10 ng ml^−1^ epidermal growth factor, 36 ng ml^−1^ hydrocortisone and 4 pg ml^−1^ triiodothyronine) in the presence of 0.5% fetal calf serum (FCS). After 1–2 days, medium was replaced by FCS-free medium. For HIF stabilization, cells were incubated for 16 h with dimethyloxalylglycine (DMOG; Sigma Aldrich) in a final concentration of 1 mM or 2-(1-chloro-4-hydroxyisoquinoline-3-carboxamido) acetate (ICA; kind gift from Prof. Dr N. Burzlaff, Department of Chemistry, University of Erlangen-Nürnberg) in a final concentration of 100 µM. In addition to ICA, dimethyl N-oxalyl-D-phenylalanine (NOFD; Sigma Aldrich) in a final concentration of 1 mM was used for FIH inhibition.

### 4.2. Chromatin immunoprecipitations

HIF-1*α* and HIF-1*β* ChIP qPCR and ChIP-seq data from human primary tubular epithelial cells were generated as described previously [40]. Antibodies used were: HIF-1*α* (rabbit polyclonal, Cay10006421, Cayman Chemicals, Ann Arbor, MI) and HIF-1*β* (rabbit polyclonal, NB100-110, Novus Biologicals, Centennial, CO). Primer sequences are available in electronic supplementary material, table S1.

### 4.3. Assay for transposase accessible chromatin sequencing (ATAC)

Cells were grown under standard culture conditions and harvested at 80% confluency. Cells were trypsinized, manually counted and 60 000 cells were directly subjected to the Omni-ATAC protocol as described by Corces *et al.* [41]. Preparation of libraries, sequencing and data analysis were conducted as described earlier [42]. To define the ATAC landscape in whole kidneys, the summit method described by Corces *et al*. was used to define the landscape of open chromatin in whole kidney tissue [43]. 15 BED files were collapsed into the whole kidney ATAC landscape via BEDOPS-software (v. 2.4.39) [44]. The following bed files from ATAC-seq of whole kidneys were provided by the Susztak laboratory and downloaded from https://cmdga.org/: DBS785VRI, DBS782VGN, DBS151FNZ, DBS799VRM, DBS285HMS, DBS929YYU, DBS521QRS, DBS042JHH, DBS020BVW, DBS267OLS, DBS738QBP, DBS329APZ, DBS743UXT, DBS835SXS, DBS908RNK.

### 4.4. ChromHMM

Human kidney ChIP-seq data for the histone marks H3K4me1, H3K4me3, H3K27ac (GSE86095) were aligned to genome hg38 and were used to annotate chromatin states using chromHMM (v. 1.11) [21,22]. Results from these analyses for chromosome 16 are available in electronic supplementary material, file S2.

### 4.5. RNA-Seq

100 bp single-end sequencing was performed on RNA samples from PTC yielding approximately 30 million reads per sample. After quality check using FastQC (v. 0.11.8; available online at: http://www.bioinformatics.babraham.ac.uk/projects/fastqc/) reads were aligned to the human reference genome (hg38) using the STAR alignment software (v. 2.6.1c) [45]. Mapped reads were used to generate a count table using the feature-counts software (v. 1.6.1) [46]. Briefly, raw reads were filtered, normalized and visualized by using R version 4.1.1. DESeq2 package v. 1.32.0 was used for logarithmic transformation of the data and for data exploration [47]. Differential expression analysis was done by using the DESeq2 lfc Shrink approach. Adjusted *p*-values were calculated using the Benjamini–Hochberg method within DESeq2. Gene annotations were added to the result files using biomaRT (v. 2.48.3) [48]. Different conditions were compared (sample size: control PTC *n* = 3 versus hypoxia *n* = 3, control PTC *n* = 7 versus DMOG *n* = 7, control PTC *n* = 3 versus ICA *n* = 3, control PTC *n* = 3 versus ICA + NOFD *n* = 3). Results from these analyses are available in electronic supplementary material, file S1.

### 4.6. Animal experiments

Animal experiments were approved by the local institutional review board and all animal experiments complied with the United Kingdom Animals Act, 1986, and associated guidelines, EU Directive 2010/63/EU for animal experiments. Experiments were approved by the local Ethics Committee of the Government of Unterfranken/Würzburg (AZ: RUF 55.2.2-2532.2-806-14). Male adult wild-type mice with C57BL/6 background were either injected intraperitoneally with the PHD-inhibitor ICA (*n* = 6; 40 mg kg^−1^ body weight; dissolved in 5% dimethyl sulfoxide and 95% 0.5 mol/LTris), the combination of ICA and the FIH-inhibitor NOFD (*n* = 6; 80 mg kg^−1^) or vehicle solution serving as control (*n* = 6). Kidneys were harvested 6 hours after injection. Total RNA from organs was isolated using the peqGOLD Total RNA Kit (VWR peqlab) according to manufacturer's instructions. Transcription of RNA to cDNA was performed using the High-Capacity cDNA Reverse Transcription Kit (Thermo Fisher Scientific).

### 4.7. Real-time polymerase chain reaction

SYBR Green-based real-time polymerase chain reaction was performed using StepOnePlus (Applied Biosystems, Foster City, CA). mRNA expression levels were normalized to 18S ribosomal RNA in mice or HPRT in hPT cells using the double delta threshold cycle (ΔΔCt) method. Primer sequences are listed in electronic supplementary material, table S1.

### 4.8. siRNA of HIF-1*α* and HIF-1*β*

The use of siRNA directed against *Drosophila* HIF (dHIF; serving as control siRNA), HIF-1*α* and HIF-1*β* has been described previously [49]. siRNAs were transfected using Lipofectamin3000 reagent (Thermo Fisher Scientific, Inc., Erlangen, Germany) with a final concentration of 40 nM. siRNA sequences are listed in electronic supplementary material, table S2.

### 4.9. Western blotting

Proteins were isolated from hPT cells or mouse kidneys using a sample buffer containing 50 mM Tris-HCl, 150 mM NaCl, 10 mM EDTA, 1% sodium deoxycholate, 0.1% SDS and 1% protease inhibitor mixture (Roche, cOmplete, EDTA-free, Mannheim, Germany) and 1% Triton X-100. Proteins were separated using NuPAGE 3–8% Tris-Acetate Protein Gels (Life Technologies/Gibco, Karlsruhe, Germany) for detection of PC1. Proteins were blotted using an iBlot 2 Dry Blotting System (Thermo Fisher Scientific, Inc., Erlangen, Germany) on to a polyvinylidene difluoride membrane (GE Healthcare Europe GmbH, Munich, Germany). Membranes were then incubated with primary anti-PC1 antibody (7E12; Santa Cruz; 1 : 1000) or primary anti-HIF-1*α* antibody (Cayman Chemicals, Ann Arbor, MI; 1 : 1000) overnight. Proteins were visualized using horseradish peroxidase-conjugated secondary antibody and ECL detection. We performed a combined densitometric analyses of all three detectable bands of PC1 (PC1-FL, PC1-NTR, and PC1-NTS), because all of these bands appeared to be upregulated upon HIF-stabilization. In addition, we were unable to dissect the bands adequately in the mouse whole kidney lysates, due to technical difficulties. Vinculin and Amido black staining were used as loading control.

### 4.10. RNAscope

Localization of mRNA was studied with the 2.5 HD Detection DAB Kit (ACD 322310; Advanced Cell Diagnostics ACD, Hayward, CA), according to the manufacturer's instructions. Hybridization signals were detected using 3,3′-diaminobenzidine. Slices were mounted with EcoMount mounting medium (Biocare Medical, EM897L) and analysed with a Leica DM6000B microscope and a Leica DFC 450C camera. The following RNAscope probe was used for detection of PKD1 mRNA: Mm-PKD1 Ref. 549151 Fa. ACD. In addition, RNAscope Negative Control Probe-DapB, Ref. 310043 served as negative control. Thereafter, PKD1 mRNA signals were analysed by setting 10 ureteric bud branches per condition as region of interest and the use of a colour deconvolution algorithm (ImageJ software v. 1.48) to dissect the different signals, followed by binarization and particle analysis to obtain the ratio of positive PKD1 area to the marked branching area.

### 4.11. Metanephric organ culture

Metanephric kidneys were dissected from embryonic mice with C57BL/6 background at embryonic day E13.0 and cultured on transparent Millicell organotypic cell culture inserts (Merck Millipore, Billerica, MA) as described previously [18]. After 24 h, medium was changed and one kidney from each embryo was maintained under control conditions, whereas the contralateral kidney was incubated with DMOG (1 mM) or ICA (100 µM) or kept under hypoxic conditions in the presence of 1% O_2_ for 24 h. Then, kidneys were fixed in paraformaldehyde (4%).

### 4.12. Statistical analysis

Data are expressed as mean ± s.e.m. The differences among groups were analysed using one-way ANOVA, followed by a Bonferroni test for multiple comparisons. Wilcoxon signed-rank test for column statistics was used for relative values. For RNA-seq data, adjusted *p*-values were calculated using the Benjamini–Hochberg method within DESeq2 and error bars represent the standard error of log_2_-fold change. *p* < 0.05 was considered statistically significant.

## Data Availability

ChIP-seq, ATAC-seq and RNA-seq data from hPTC are available at GEO (accession no. GSE227849). ChIP-seq data from HKC-8, 786-0 and RCC4 cells were obtained from GEO (GSE120887 and GSE67237). RNA-seq results for 786-0 cells were downloaded from GEO (GSE153711 and GSE67237). RNA-seq data from HKC-8 cells exposed to 21% or 0.1% O_2_ for 16 h was from GSE120886 and reanalysed to map against hg38. Human kidney ChIP-seq data for the histone marks H3K4me1, H3K4me3 and H3K27ac were downloaded from GSE86095. ATAC-seq data of whole kidneys were provided by the Susztak laboratory and downloaded from https://cmdga.org/ as mentioned above. The data are provided in electronic supplementary material [50].
